# How does women’s empowerment relate to antenatal care attendance? A cross-sectional analysis among rural women in Bangladesh

**DOI:** 10.1186/s12884-023-05737-9

**Published:** 2023-06-13

**Authors:** Solis Winters, Helen O. Pitchik, Fahmida Akter, Farzana Yeasmin, Tania Jahir, Tarique Md. Nurul Huda, Mahbubur Rahman, Peter J. Winch, Stephen P. Luby, Lia C. H. Fernald

**Affiliations:** 1 School of Public Health, University of California, Berkeley, 2121 Berkeley Way West, Berkeley, CA 94720 USA; 2 Environmental Interventions Unit, Infectious Diseases Division, icddr,b, Dhaka, 1212 Bangladesh; 3 College of Medicine, Nursing, & Health Sciences, University of Galway, Galway, Ireland; 4 Department of Public Health, College of Public Health and Health Informatics, Qassim University, Al Bukairiyah, 52741 Saudi Arabia; 5 Department of International Health, Bloomberg School of Public Health, Johns Hopkins University, Baltimore, MD USA; 6 Division of Infectious Diseases and Geographic Medicine, Stanford University, Stanford, CA USA

**Keywords:** Maternal and child health, Antenatal care, Pregnancy, Women’s empowerment, Decision-making power, Freedom of movement, Control over assets, Low- and middle- income countries, Bangladesh

## Abstract

**Background:**

In South Asia, roughly half of women attend at least four antenatal care visits with skilled health personnel, the minimum number recommended by the World Health Organization for optimal birth outcomes. A much greater proportion of women attend at least one antenatal care visit, suggesting that a key challenge is ensuring that women initiate antenatal care early in pregnancy and continue to attend after their first visit. One critical barrier to antenatal care attendance may be that women do not have sufficient power in their relationships, households, or communities to attend antenatal care when they want to. The main goals of this paper were to 1) understand the potential effects of intervening on direct measures of women’s empowerment—including household decision making, freedom of movement, and control over assets—on antenatal care attendance in a rural population of women in Bangladesh, and 2) examine whether differential associations exist across strata of socioeconomic status.

**Methods:**

We analyzed data on 1609 mothers with children under 24 months old in rural Bangladesh and employed targeted maximum likelihood estimation with ensemble machine learning to estimate population average treatment effects.

**Results:**

Greater women’s empowerment was associated with an increased number of antenatal care visits. Specifically, among women who attended at least one antenatal care visit, having high empowerment was associated with a greater probability of ≥ 4 antenatal care visits, both in comparison to low empowerment (15.2 pp, 95% CI: 6.0, 24.4) and medium empowerment (9.1 pp, 95% CI: 2.5, 15.7). The subscales of women’s empowerment driving the associations were women’s decision-making power and control over assets. We found that greater women’s empowerment is associated with more antenatal care visits regardless of socioeconomic status.

**Conclusions:**

Empowerment-based interventions, particularly those targeting women’s involvement in household decisions and/or facilitating greater control over assets, may be a valuable strategy for increasing antenatal care attendance.

**Trial registration:**

ClinicalTrials.gov Identifier: NCT04111016, Date First Registered: 01/10/2019.

**Supplementary Information:**

The online version contains supplementary material available at 10.1186/s12884-023-05737-9.

## Background

Approximately 6,700 newborns die every day, accounting for roughly half of all child deaths under five years old; the majority of these deaths are in low- and middle-income countries (LMICs) and around 75 percent occur within the first week of life [1]. Sub-Saharan Africa and Southern Asia have the highest neonatal mortality rates globally; a child born in these regions is 10 times more likely to die in the first month than a child born in a high-income country [1]. Preterm birth, intrapartum-related complications, infections, and birth defects are the leading causes of neonatal deaths. In LMICs, around half of babies born before 32 weeks die, and many who survive face lifelong disabilities [1, 2].

Antenatal care is associated with newborn survival and health [3]. In 2016, the World Health Organization issued new comprehensive guidelines on antenatal care for a positive pregnancy experience, which increased the minimum recommended number of antenatal care contacts with health professionals from four to eight based on evidence that a reduced number of antenatal care visits was associated with more perinatal deaths [4–7]. This is consistent with a survival analysis of data from 57 LMICs, which found a 51% lower risk of neonatal mortality among women who attended ≥ 4 antenatal care visits and a 26% lower risk among women who had at least one antenatal care visit in the first trimester [8]. Inadequate antenatal care is also associated with adverse birth outcomes, which in addition to increased risk of death, can have lifelong effects on child development [9–12]. This may be at least partly because attending antenatal care visits promotes skilled birth attendance and institutional delivery [13], and ensures receipt of core healthcare services including blood pressure measurement, tetanus toxoid vaccination, urine testing, iron tablet supplementation, body weight measurement, and counseling about danger signs [5, 7, 14, 15].

In South Asia, only 49% of women attend at least four antenatal care visits, while 80% attend at least one, suggesting that a key challenge is ensuring that women initiate antenatal care early in pregnancy and continue attending after their first visit [16]. Lack of accessible and affordable antenatal care services and low health insurance coverage create substantial barriers to care for certain segments of the population, including women with low household income, wealth, and/or education, and women in rural areas [17–27]. Disempowerment and low status of women due to persistent patriarchal norms and traditional gender roles also create additional barriers to antenatal care attendance, if women do not have sufficient power in their relationships, households, or communities to attend antenatal care visits when they want to [19, 20, 24, 28, 29].

### Potential pathways between women’s empowerment and antenatal care

Women’s empowerment is a complex, multidimensional concept that varies across cultures, and most often refers to a woman’s ability to make life choices [24, 30–33]. Three inter-related dimensions comprise women’s empowerment [31, 32]. First, having access to material, human, and social resources and institutional environments that allow one to freely make a choice (e.g., education, social status, wealth, health care). Second, having agency, or the ability to define one’s goals and act upon them (e.g., decision-making power, bargaining power, mobility in the public domain). Third, achieving equality at the individual, household, and societal level (e.g., financial autonomy, marriage and gender equality, labor market participation).

Women’s empowerment plays an important role in newborn health and development, both through greater access to resources and agency [30, 32, 33]. This may be due to greater use of contraception and maternal health services and higher rates of exclusive breastfeeding among more empowered women [19, 34–36]. A large body of evidence shows that empowerment measures related to women’s access to resources, including education, household wealth, and employment status, are positively associated with increased antenatal care attendance [20, 21, 24, 25, 28, 29, 37]. However, fewer studies have examined the link between antenatal care attendance and measures of women’s empowerment that focus specifically on agency (e.g., decision-making power, bargaining power, mobility in the public domain). We refer to these measures as direct measures of empowerment.

A few papers in South Asia and Sub-Saharan Africa show positive associations of varying magnitudes between decision-making power, freedom of movement, and control over assets and antenatal care attendance [19, 20, 24, 28, 29]. For example, in a nationally representative sample of women of reproductive age in Bangladesh, participation in household decision making, freedom of choice in contraception or going out alone, and involvement in economic activities were each associated with a 2–3 percentage point higher probability of receiving any antenatal care [20]. Women in North India with greater freedom of movement attended more antenatal care visits [19], as did women in Ethiopia and Eritrea who were involved in household decision making [24, 28, 29].

However, the body of evidence around direct measures of women’s empowerment and antenatal care attendance is still relatively limited, especially among rural populations. While positive associations between decision-making power, freedom of movement, and control over assets and antenatal care attendance have been found in some contexts, it is still not clear which measures of women’s empowerment have the greatest potential for improving antenatal care attendance in rural Bangladesh. Moreover, the study in Bangladesh looked at direct measures of empowerment but did not examine the combined effect of these separately from indirect measures like education [20]. Additionally, these papers employ parametric regression models, which, if misspecified, can result in biased estimates. Sources of heterogeneity have also not been explored, which may have important implications for targeting of interventions.

### Study aims

The main goal of this paper is to understand the potential effects of intervening on direct measures of women’s empowerment—including household decision making, freedom of movement, and control over assets—on antenatal care attendance in a rural population of women in Bangladesh. We approach this question through a population-based, counterfactual framework. More specifically, we estimate antenatal care attendance in the population after setting women’s empowerment to different hypothetical scenarios of low, medium, and high empowerment. We also analyze differential associations by socioeconomic status, to better understand whether women’s empowerment operates differently among populations with varying access to resources. Our main hypothesis was that there would be a gradient relationship between women’s empowerment level and antenatal care attendance: i.e., the probability of attending antenatal care would be highest under the hypothetical scenario in which all women have high empowerment and lowest if everyone had low empowerment, for both the aggregate empowerment score and for each dimension separately. We also hypothesized that there would be differential associations by socioeconomic status: specifically, that the associations would be largest among households with higher socioeconomic status.

### Setting: rural Bangladesh

A lower-middle-income country with a per capita GDP of 1,962 USD, Bangladesh currently has the eighth highest number of newborn deaths and the seventh highest number of preterm births globally (World Bank, 2020; World Health Organization, 2018). Antenatal care attendance is below the average rate for South Asia, with 75% of pregnant women attending at least one antenatal care visit by skilled health personnel and 37% attending at least four visits in 2019 [16]. Less than 18% of women received “quality antenatal care”, defined per Bangladesh country guidelines as attendance of ≥ 4 antenatal care visits (with ≥ 1 visit from a skilled health personnel) and receipt of core services [38].

Bangladesh has made remarkable progress on women’s empowerment and gender equality over the last 30 years, a time in which maternal mortality and fertility rates have rapidly declined, the gender gap in primary and secondary education has closed, and the proportion of female wage workers in non-agricultural employment has steadily increased [39]. Despite these notable gains, certain patriarchal norms and practices persist and have resulted in powerlessness and discrimination of many women in other aspects of life [39]. Violence against women is still pervasive, with 73% of ever married women reporting having experienced violence by their husband at least once in their lifetime and 55% experiencing violence in the last 12 months [40]. The prevalence of child marriage in Bangladesh is also high, with 59% of girls getting married before 18 [41].

## Methods

### Data

We analyzed baseline data collected for the RINEW-G (Research on Integration of Nutrition, Early Childhood Development and WASH through the government healthcare system) study in Bangladesh. The study was registered at ClinicalTrials.Gov (NCT04111016) on 01/10/2019 [42]. The intervention was implemented across the sub-district of Chatmohar. All the unions of Chatmohar sub-district were selected except for the Sadar Upazila, which was excluded because it is an urban center and differs in terms of socioeconomic status, demographic indicators, and health care relative to the other 11 rural unions.

To identify eligible households for the baseline survey, a team of 15 enumerators visited every household in the Chatmohar sub-district and screened each household based on the following inclusion criteria: 1) mother or caregiver of a child 6–24 months old, 2) living in 11 rural unions of Chatmohar, and 3) planning to reside in Chatmohar for at least one year. Mothers and children with impaired cognitive development, hearing, vision, and/or speech were excluded. 6,581 households in 255 villages across the 11 unions were identified. A multistage sampling strategy was then used to select households for the baseline survey. More specifically, first 109 villages were allocated across the 11 unions according to population size in the most recent census, then proportionate population sampling was used to select specific villages within the 11 unions, and finally stratified random sampling was used to select households within the 109 selected villages.

Baseline data collection began on April 18, 2019 and was completed by June 22, 2019. Data were collected through household visits. The baseline survey includes a rich set of demographic, socioeconomic, health, and empowerment variables for 1635 caregivers with children 6–24 months old. 1609 biological mothers were included in the analysis. 26 grandmothers and aunts were excluded to ensure that antenatal care attendance was recent and over the same time period. The study hypotheses, variables, and planned analyses were preregistered on Open Science Framework after data collection but before primary analysis [43].

### Measurement of antenatal care visits

The mother self-reported the number of antenatal care visits attended during her most recent pregnancy. These were categorized into three groups based on Bangladesh country guidelines, which recommend at least four antenatal care (ANC) visits: no ANC, 1–3 ANC visits, and ≥ 4 ANC visits. Generated binary variables for all pairwise comparisons (≥ 4 vs. 1–3 ANC, no ANC vs. 1–3, and ≥ 4 vs. no ANC).

### Measurement of women’s empowerment

While there is some consensus around the broad dimensions of women’s empowerment, the abstract and multidimensional nature of women’s empowerment makes it difficult to measure, with significant debate about the appropriate level of aggregation, specific criteria that should be included, and relative importance of each dimension [32, 44]. Though no standard, universally accepted measure exists, a couple of well-known indices that have been developed to measure women’s empowerment in different contexts [30, 32, 44, 45].

We generated an aggregate score for women’s empowerment following the approach used for the Women’s Empowerment in Agriculture Index (WEAI), which was developed to measure women's empowerment, agency, and inclusion in the agriculture sector [45]. Mothers answered 15 survey questions related to three main domains—control over assets, freedom of movement, and household decision making. Responses indicative of greater empowerment were given a higher number. A weighted sum score was computed, where each domain was given equal weight. The score was re-scaled to values between 0 and 100 (Table S1). For easier interpretation, the empowerment score was categorized into high, medium, and low empowerment, representing the top 25%, middle 50%, and bottom 25% of the distribution, respectively.

In addition to the aggregate measure, three subscales were generated for each of the three domains. The decision-making power subscale is the sum of answers to answers to 7 survey questions about women’s involvement in household decisions, where higher numbers indicate greater decision-making power. The freedom of movement subscale is the sum of answers to 3 questions about women’s ability to go to the market, village, or family/friends’ homes, where higher scores represent greater freedom of movement. The control over assets subscale is the sum of answers to 5 survey questions indicating women’s control over earnings and assets, where higher scores indicate greater control over assets. All subscales were categorized into high, medium, and low in the same way as for the overall empowerment score (Table S1).

To assess the robustness of our findings to different ways of measuring women’s empowerment, we generated another set of indices following the approach used for the Survey-based Women’s emPowERment (SWPER) index [44]. The SWPER applies principal component analysis to extract the most important components from a list of items related to women’s empowerment, including questions around attitude to violence, social independence, and decision making. We conducted principal components analysis with the same list of items included in the primary empowerment measures described above (Table S1).

### Covariates

Individual and household characteristics known to be associated with antenatal care attendance and women’s empowerment were included as covariates. These include women’s age (years), women’s depressive symptoms (ranging from 0 to 60 on the Center for Epidemiologic Studies Depression Scale (CESD) [46]), women’s education (years), the education differential between the woman and her partner (years), child’s age (months), number of members in the household, number of children under 15 in the household, and household wealth (the first principal component generated from applying principal components analysis to 43 variables representing asset ownership).

### Subgroups

Household socioeconomic status was estimated with the water/sanitation, assets, maternal education, and income (WAMI) index, which is composed of four components: access to improved water and sanitation, wealth measured by a set of eight assets, maternal education, and monthly household income (Table S2) [47]. The score ranges from 0 to 32. Households were categorized into high (> = 21), medium (> = 17 & < 21), and low (< 17) socioeconomic households based on terciles of the WAMI [47].

Subgroup analyses were also conducted for high-risk subgroups, including adolescent women, women with older children, women who live with in-laws, and women with high depressive symptoms. Adolescent women included all women who gave birth before age 20. To classify women with older children, we used a proxy variable indicating presence of more than one child under 15 in the household. Women who live with in-laws was also classified using a proxy variable indicating the presence of any adults in the household aside from the woman and her husband. Finally, women with high depressive symptoms included all women in the top quartile of the depressive symptoms scale, which includes scores greater than 18.

### Statistical analysis

#### Primary analyses

We estimated average treatment effects, or marginal risk differences, to compare antenatal care attendance across different levels of women’s empowerment. We analyzed three hypothetical scenarios in which we assigned high, medium, or low levels of women’s empowerment. More specifically, we define the following target parameters:

$${ATE}_{high-low}= E\left[Y\left(high\right)-Y\left(low\right)\right]= {E}_{w}[E\left(Y|A=high, W\right)-E\left(Y|A=low, W\right)]$$

$${ATE}_{high-med}= E\left[Y\left(high\right)-Y\left(medium\right)\right]= {E}_{w}[E\left(Y|A=high, W\right)-E\left(Y|A=medium, W\right)]$$

$${ATE}_{med- low}= E\left[Y\left(medium\right)-Y\left(low\right)\right]= {E}_{w}[E\left(Y|A=medium, W\right)-E\left(Y|A=low, W\right)]$$

where $Y\left(a\right)$ is the counterfactual outcome indicating antenatal care attendance ($Y$) when women’s empowerment ($A$) is set to $a$ = high, medium, or low, defined as the bottom 25%, middle 50%, and top 25% of the sample distribution, respectively. Four measures of antenatal care attendance ($Y$) were incorporated as outcomes, including all pairwise comparisons of the categorical antenatal care measure (≥ 4 vs. 1–3 ANC, no ANC vs. 1–3, and ≥ 4 vs. no ANC) and the total number of antenatal care visits. ${ATE}_{high-low}$ represents the difference in mean or proportion of antenatal care if the whole population had high empowerment compared to if the whole population had low empowerment, averaging over the distribution of covariates $W$. ${ATE}_{high-med}$ and ${ATE}_{med- low}$ have the same interpretation but with different exposure conditions.

Average treatment effects were estimated using targeted maximum likelihood estimation (TMLE), a doubly robust, two-stage estimation strategy, which produces a well-defined and efficient substitution estimator. TMLE takes the initial estimate of the outcome regression, $\widehat{E}\left(Y\right|A, W),$ as well as the estimated propensity score, $\widehat{P}(A=1| W)$, to produce an updated estimate, ${\widehat{E}}^{*}\left(Y\right|A, W),$ that is targeted to the parameter of interest. TMLE is unbiased if either the outcome regression or the propensity score is consistently estimated and is asymptotically efficient when both are consistently estimated. TMLE also has the flexibility to incorporate ensemble machine learning, which can help minimize bias from misspecified parametric regressions [48, 49].

TMLE with ensemble machine learning was conducted using the *tmle3* package in R [50]. The library of algorithms used to estimate the outcome regression included logistic regression, Bayesian logistic regression, LASSO, ridge regression, elastic net, random forest, extreme gradient boosting, and highly adaptive LASSO. The propensity score was estimated with LASSO, ridge regression, elastic net, random forest, and extreme gradient boosting. Missing covariates were median-imputed, and indicators for the imputed values were also included in the models. Standard errors were clustered at the village level.

#### Subgroup analyses

Stratified average treatment effects were estimated for each tercile of the WAMI index distribution in the sample, and for high-risk subgroups, including adolescent women, women with other children under 15 in the household, women with in-laws in the household, and women with high depressive symptoms scores.

#### Robustness checks

Primary results were re-analyzed using empowerment indices constructed with principal components analysis, following the approach used for the SWPER [44]. As an additional robustness check, average treatment effects for the overall empowerment index were also estimated using parametric G-computation [49].

## Results

### Study population

The study population includes 1609 mothers with children 6–24 months old. Seventeen observations missing education differential and 1 observation missing women’s depressive symptoms were median-imputed.

At the time of data collection, most women were in their twenties. Measures of household socioeconomic status, such as income, wealth, education, and water/sanitation, were similar across empowerment groups. However, the education differential between women and their husbands was greater for higher levels of empowerment. More empowered women had fewer children and in-laws in the household than women with lower empowerment. On average, women attended 3 (SD: 2.7) antenatal care visits, with about 34% of women attending ≥ 4 visits. Women with higher empowerment attended more visits than those with lower empowerment (Table 1).

**Table 1:** Sample characteristics, overall and by empowerment level

	**Full sample**	**Low empowerment**	**Medium empowerment**	**High empowerment**
n	1609	402	788	419
Women's age	25.8 (5.5)	24.9 (5.7)	25.6 (5.4)	27.1 (5.1)
Women's depressive symptoms	13.8 (8.9)	14.2 (9.0)	13.7 (8.8)	13.5 (9.0)
Women's education	6.6 (3.5)	6.3 (3.2)	6.7 (3.6)	6.8 (3.5)
Education differential	1.1 (3.8)	0.9 (3.7)	0.8 (3.8)	1.8 (4.0)
Wealth	0.0 (2.4)	-0.0 (2.4)	0.0 (2.5)	-0.1 (2.4)
Monthly household income ($)	172.6 (163.5)	162.7 (130.9)	177.5 (189.4)	173.0 (136.7)
Improved water	1609 (100%)	402 (100%)	788 (100%)	419 (100%)
Improved sanitation	1100 (68%)	277 (69%)	540 (69%)	283 (68%)
WAMI	18.6 (4.4)	18.3 (4.4)	18.5 (4.5)	18.8 (4.2)
Number of members	5.2 (1.9)	5.7 (2.0)	5.2 (1.9)	4.7 (1.6)
Number of in-laws	1.3 (1.9)	2.0 (2.1)	1.3 (1.9)	0.72 (1.5)
Number of children < 15	1.9 (0.8)	1.8 (0.8)	1.8 (0.8)	2.01 (0.8)
Youngest child's age, months	13.5 (5.3)	13.2 (5.1)	13.5 (5.4)	13.82 (5.5)
Empowerment score	46.0 (17.1)	24.1 (7.6)	45.7 (6.5)	67.45 (8.4)
Number antenatal care visits	3.1 (2.65)	2.6 (2.3)	3.1 (2.7)	3.59 (2.8)
No antenatal care	218 (14%)	68 (17%)	108 (14%)	42 (10%)
1–3 antenatal care visits	841 (52%)	229 (57%)	413 (52%)	199 (48%)
≥ 4 antenatal care visits	550 (34%)	105 (26%)	267 (34%)	178 (43%)

Data are mean(SD) or n(%). Low, medium, and high empowerment categories are equal to the bottom 25%, middle 50%, and top 25% of the distribution of the empowerment score, respectively

### Overall empowerment

**Fig. 1:**
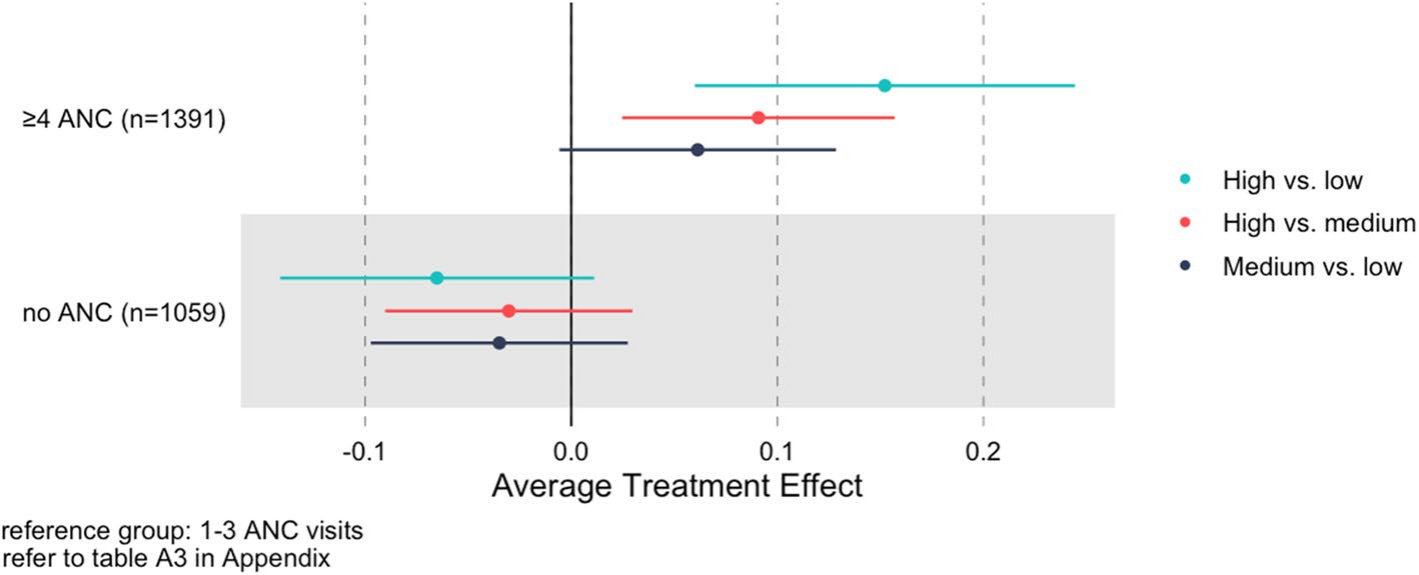
Population average treatment effects of overall empowerment for all pairwise comparisons of the categorical antenatal care (ANC) measure, full sample

Among women who attended at least one antenatal care visit, having high empowerment was associated with a greater probability of ≥ 4 antenatal care visits, both in comparison to low empowerment (ATE: 15.2 percentage points (pp), 95% CI: 6.0, 24.4) and medium empowerment (9.1 pp, 95% CI: 2.5, 15.7). These represent relative increases in probability of attending at least four visits of 45% and 26%, respectively. Having medium empowerment was also associated with a greater probability of ≥ 4 antenatal care visits compared to having low empowerment (6.1 pp, 95% CI: -0.6, 12.8) (Fig. 1). These results indicate a positive gradient between women’s empowerment and antenatal care attendance: that is, each additional increase in empowerment level is associated with a greater probability of attending ≥ 4 antenatal care visits. Notably, the magnitude of the association between high and medium empowerment is larger than the association between medium and low empowerment. Similar estimates for the association between empowerment and probability of attending ≥ 4 antenatal care visits were also found when compared to women with no antenatal care visits (Table S3).

Among women who had less than four antenatal care visits, higher levels of empowerment were associated with a lower probability of no antenatal care (high vs low ATE: -6.5 pp (95% CI -14.1, 1.1); high vs medium ATE: -3.0 pp (95% CI: -9.0, 3.0); medium vs low ATE: -3.5 pp (95% CI: -9.7, 2.7)) (Fig. 1).

These findings are consistent with those looking at the number of antenatal care visits. Among women who attended at least one antenatal care visit, high empowerment was associated with 1 additional antenatal care visit (95% CI: 0.7, 1.5) compared to low empowerment and 0.5 additional visits (95% CI: 0.2, 0.9) compared to medium empowerment. Medium empowerment was also associated with a higher number of antenatal care visits compared to low empowerment (0.5 visits, 95% CI: 0.3, 0.8). Stratified estimates were similar across all analyses (Table S4).

**Fig. 2:**
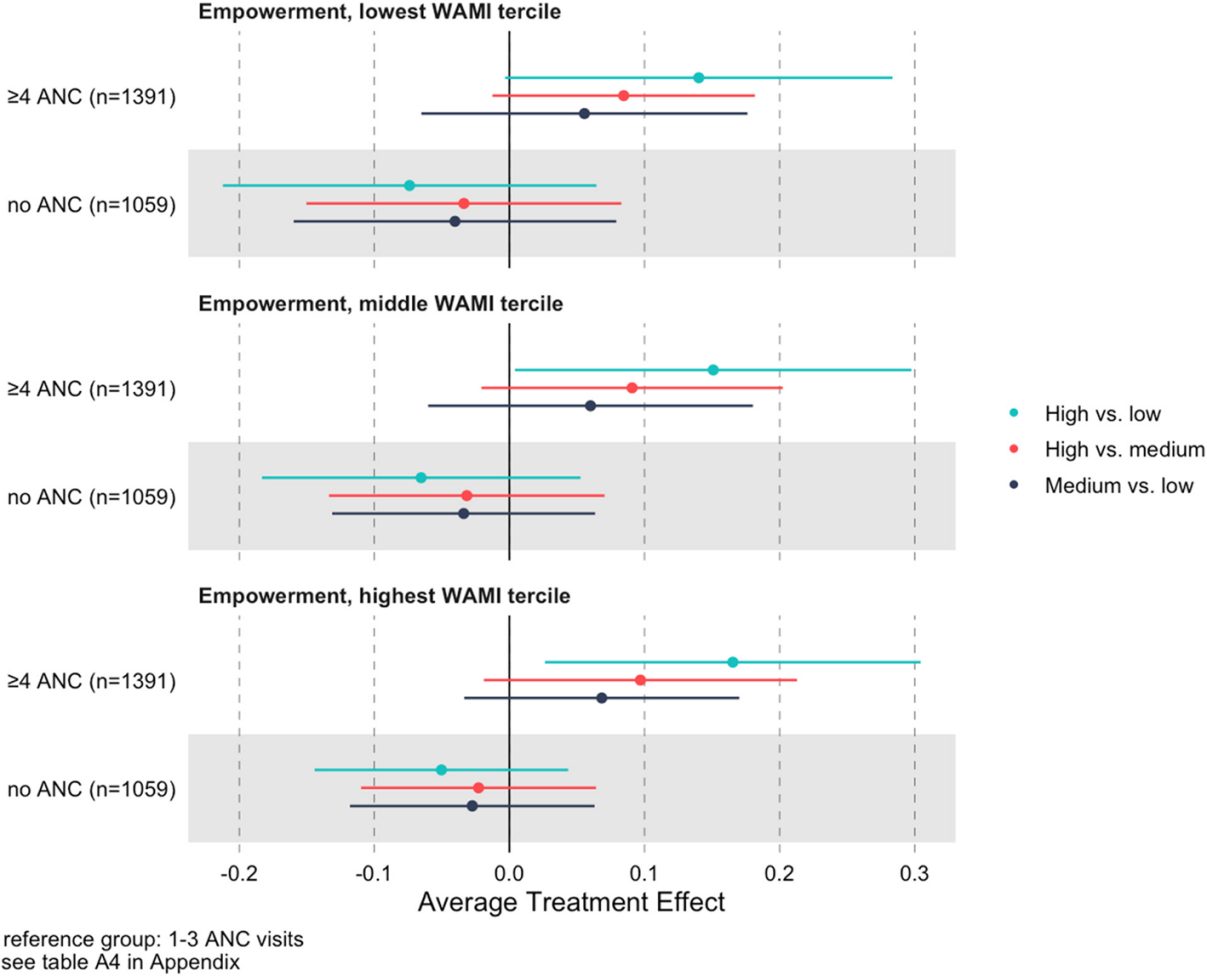
Population average treatment effects of overall empowerment for all pairwise comparisons of the categorical antenatal care (ANC) measure, stratified by WAMI tercile

To assess whether the magnitude of these differences varies by socioeconomic status, we estimated stratified average treatment effects for terciles of the WAMI. The estimates do not vary across strata and are of similar magnitude compared to the full sample estimates, suggesting an association with antenatal care independent of socioeconomic status (Fig. 2) (Table S5).

### Empowerment subscales

To better understand the mechanisms driving the aggregate associations, we estimated differences in antenatal care attendance associated with each of the three dimensions of empowerment separately – decision-making power (Table S6), freedom of movement (Table S7), and control over assets (Table S8).

**Fig. 3:**
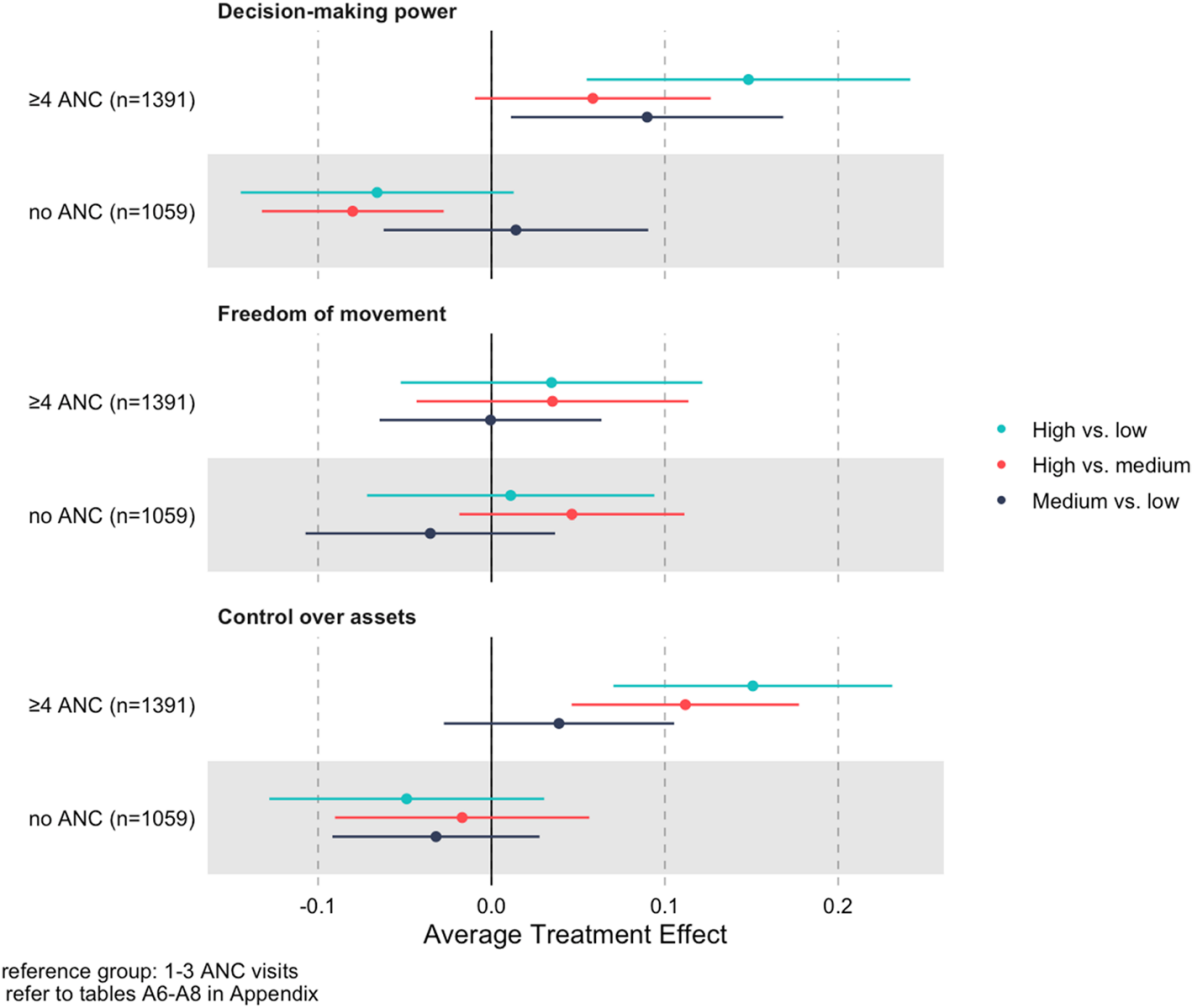
Population average treatment effects of the empowerment subscales for all pairwise comparisons of the categorical antenatal care (ANC) measure, full sample

Among women who attended at least one antenatal care visit, high and medium decision-making power were associated with 15 pp (95% CI: 5.5, 24.2) and 9 pp (95% CI: 1.1, 16.8) higher probability of ≥ 4 antenatal care visits, respectively, compared to low decision-making power. High control over assets was associated with a 15 pp (95% CI: 7.0, 23.1) higher probability of attending ≥ 4 antenatal care visits compared to low control over assets, and an 11 pp (95% CI: 4.6, 17.7) higher probability compared to medium control over assets (Fig. 3). These estimates are similar in magnitude to those from the overall empowerment scale. The associations between freedom of movement and antenatal care attendance are much lower in magnitude relative to the estimates using the overall empowerment scale: among women who attended at least one antenatal care visit, high freedom of movement was associated with 3.5 pp increase in ≥ 4 antenatal care visits compared to medium (95% CI: -5.2, 12.2) and low (95% CI: -4.3, 11.4) freedom of movement, respectively (Fig. 3).

The direction and magnitude of these associations are consistent with average treatment effects on the total number of antenatal care visits (Tables S12, S13, S14), and estimates stratified by WAMI tercile (Figures S1, S2, S3, Tables S9, S10, S11).

### Subgroup analyses

To assess whether there were any differential associations between empowerment and antenatal care attendance, we estimated stratified average treatment effects for specific risk factors. We found little variation in effect sizes between adolescent and adult women, women with and without in-laws or other children under 15 in the household, or women at risk for depression (Figures S4, S5, S6, S7).

### Robustness checks

Average treatment effects estimated with parametric G-computation are consistent with findings using targeted maximum likelihood estimation (Table S15).

To see if our results were robust to changes in how empowerment is measured, we replicated all the results using indices constructed with principal components analysis, following the approach used for the SWPER. The first three components were kept, representing decision-making power, freedom of movement, and control over assets, respectively (Table S16, Figure S8). The findings are consistent with our domain analyses, which show positive and significant associations between decision-making power and control over assets and antenatal care, but no association with freedom of movement (Tables S17, S18, S19).

## Discussion

Higher women’s empowerment was associated with a greater likelihood of continuing antenatal care in our study setting in rural Bangladesh. Among women who attended at least one antenatal care visit, we found that high empowerment was associated with a greater probability of attending ≥ 4 antenatal care visits compared to low empowerment, as well as compared to medium empowerment. These associations were largest for women’s decision-making power and control over assets domains. There was no association between freedom of movement and antenatal care attendance. The associations between women’s empowerment, overall and for each dimension separately, were similar across all terciles of the WAMI index, suggesting that empowerment is positively associated with antenatal care independent of socioeconomic status.

Our finding that higher empowerment was associated with greater antenatal care attendance is consistent with the existing, limited evidence in LMIC. A few papers, including one in Bangladesh, have found positive associations between measures of women’s empowerment and antenatal care attendance [20, 24, 28, 29]. In Bangladesh, participation in household decision making, freedom of choice in contraception or going out alone, and involvement in economic activities together with completion of secondary education was associated with an estimated 24% higher likelihood of any antenatal care compared to no education and no participation in decisions, freedom of choice/movement, or involvement in economic activities [20]. Studies in Ethiopia, India, and Eritrea also found positive associations between measures of empowerment, including decision-making power, freedom of movement, and low tolerance of violence, and the number of antenatal care visits attended [24, 28, 29]. While these studies show that women’s empowerment is an important facilitator of antenatal care attendance in general, they vary widely in which dimensions of empowerment are studied, the specific list of items asked within each dimension, and the methods are chosen to create empowerment-based indices. Furthermore, there is a range of antenatal care outcomes studied, including any antenatal care, at least four antenatal care visits, or the total number of antenatal care visits. Our study contributes to the literature by combining three commonly studied dimensions of women’s empowerment – decision-making power, freedom of movement, and control over assets—into one composite index to determine how empowerment across multiple dimensions is associated with antenatal care attendance. We also analyzed a few different measures of antenatal care attendance, including differences between categories of antenatal care (no antenatal care, 1–3 antenatal care visits, and ≥ 4 antenatal care visits) and by the total number of antenatal care visits.

The association between high empowerment and high antenatal care attendance was driven primarily by greater decision-making power and control over assets. We found no association between freedom of movement and antenatal care attendance, which may mean this is not a limiting constraint to attending antenatal care in this population. These findings contribute to inconclusive evidence around the relative importance of specific dimensions of women’s empowerment on antenatal care attendance. In Bangladesh, decision-making power and control over assets were positively associated with any antenatal care use and frequency of antenatal care, but the association between freedom of choice/movement was no longer significant when looking at the frequency of antenatal care [20]. Other evidence from Ethiopia, Eritrea, Pakistan, and Albania show positive associations between decision-making power and antenatal care attendance [24, 28, 29, 51]. In India, however, freedom of movement was positively associated with quality antenatal care while decision-making power and control over finances were not [19]. Mixed findings may be due to differences in how the dimensions were measured. It is possible that our null finding on freedom of movement is due at least in part to low variability in the index, since it is only based on three items. Certain aspects of empowerment may also be more or less important in different contexts and cultures. Our findings are consistent with the findings from Bangladesh [20].

We also found similar associations between women’s empowerment and antenatal care attendance across different strata of socioeconomic status, age at childbirth, parity, presence of in-laws in the household, and depressive symptoms. These findings suggest that low empowerment may be a barrier to antenatal care attendance for all women in these communities, independent of risk status. We may also see less heterogeneity due to the fact that our sample consists of a rural population of women concentrated in one sub-district.

This study provides a few important strengths. First, we test how a hypothetical intervention targeting women’s empowerment could change the probability of reaching key antenatal care targets through a counterfactual framework. Our combined measure of women’s empowerment only includes direct measures of empowerment that can be influenced once women are already of reproductive age, unlike many other indices that include maternal education, which requires intervention long before a woman becomes pregnant. Moreover, we assessed differential associations between women’s empowerment and antenatal care based on socioeconomic status and explored sources of heterogeneity across key risk factors of antenatal care attendance, including mother’s age, presence of in-laws, parity, and depressive symptoms. In addition, while most studies looking at women’s empowerment and antenatal care use national-level data, we focus on a rural population of women, who have disproportionately lower antenatal care attendance and higher levels of poverty. Finally, we use a rigorous methodological approach, estimating average treatment effects using targeted maximum likelihood estimation with ensemble machine learning, which produces less biased and more efficient estimates relative to traditional parametric models.

This study has several limitations. Most importantly, this is an observational, cross-sectional analysis, and while we controlled for well-known and important confounders, it is impossible to eliminate all unmeasured sources of confounding and therefore we are unable to isolate the causal effect of women’s empowerment on antenatal care attendance. Second, women’s empowerment is a multidimensional, abstract, and complex concept that is extremely difficult to measure in practice and hard to categorize into distinct domains. What it means to be empowered is very subjective and can vary widely based on context and culture. To help address this concern, we studied dimensions of empowerment that have been previously studied in the context of Bangladesh. Additionally, we used self-reported survey data which may have some imbedded bias due to social desirability and/or inaccurate recall. That being said, most other studies also rely on self-reported measures of antenatal care and women’s empowerment given challenges accessing individual health record data in low-income countries and the inherent difficulties of measuring a complex and abstract concept. To help mitigate potential bias due to length of recall or differential recall by empowerment status, we also restricted our sample to mothers who were pregnant in the last two years. Finally, these data are from one rural sub-district in Bangladesh so they may not be generalizable to urban areas in Bangladesh or other LMIC contexts.

These findings have important implications for government and community stakeholders in LMICs working to reach key antenatal care and maternal and child health targets. A number of economic empowerment interventions, including microfinance for poor women and girls, life skills and vocational training, graduation programs, and cash transfers to female beneficiaries, have found positive impacts on women’s access to economic resources and decision-making power [52–54]. While no research to our knowledge has experimentally evaluated the effect of empowerment-based interventions on antenatal care attendance directly, there have been some positive impacts on use of contraception and family planning and on child nutrition, lending greater confidence to their potential effect on maternal health services [55–57]. This evidence, paired with our findings, lends confidence to the potential success of incorporating empowerment-based components into interventions to increase antenatal care attendance in rural LMIC communities.

## Conclusions

Our study leveraged data on 1609 mothers in rural Bangladesh and targeted maximum likelihood estimation to evaluate the association between direct measures of women’s empowerment, including decision-making power, freedom of movement, and control over assets, and antenatal care attendance. We found a gradient relationship between women’s empowerment and antenatal care attendance among those who initiated antenatal care: i.e., the difference in the probability of attending ≥ 4 antenatal care visits was greatest between high and low empowerment levels (15.2 pp; 95% CI: 6.0, 24.4), followed by high vs. medium empowerment (9.1 pp; 95% CI: 2.5, 15.7), and lowest between medium vs. low empowerment (6.1 pp, 95% CI: -0.6, 12.8). These associations were driven primarily by decision-making power and control over assets, and were consistent across socioeconomic status groups. These findings suggest that incorporating empowerment-based components into interventions may be a promising strategy for improving antenatal care attendance, especially given their success in addressing other health and nutrition outcomes. In particular, interventions targeting women’s involvement in household decisions and/or facilitating greater control over assets may be especially valuable, even in families with fewer resources.

## Supplementary Information

**Additional file 1.** 

## Data Availability

The data that support the findings of this study are available from icddr,b but restrictions apply to the availability of these data, which were used under license for the current study, and so are not publicly available. Data are however available from the authors upon reasonable request and with permission of icddr,b.
