# Supplementary material for: How does women’s empowerment relate to antenatal care attendance? A cross-sectional analysis among rural women in Bangladesh
Source: BMC Pregnancy Childbirth. 2023 Jun 13;23:436. doi: 10.1186/s12884-023-05737-9 (PMC10262442; doi:10.1186/s12884-023-05737-9)
Supplement: Supplementary file 1 — Additional file 1. [file 12884_2023_5737_MOESM1_ESM.docx]

**Supplementary Material**

Table S1. Components of the aggregate empowerment index and item weights

| **Domain** | **Indicator** | **Code** | **Weight** |
| --- | --- | --- | --- |
| **Decision Making** | In your household, who usually makes decisions about food purchased for the household? | 1-mother involved, 0-otherwise | 1/21 |
|  | In your household, who usually makes decisions about how long your children stay in school? | 1-mother involved, 0-otherwise | 1/21 |
|  | In your household, who usually makes decisions about health expenses for your children (including travel to clinic)? | 1-mother involved, 0-otherwise | 1/21 |
|  | In your household, who usually makes decisions about your own health care (doctors’ visits, medicine)? | 1-mother involved, 0-otherwise | 1/21 |
|  | In your household, who usually makes decisions about household improvements (e.g., toilets)? | 1-mother involved, 0-otherwise | 1/21 |
|  | In your household, who usually makes decisions about household goods (e.g., food containers and soap)? | 1-mother involved, 0-otherwise | 1/21 |
|  | In your household, who usually makes decisions about items for your children (e.g., toys, books)? | 1-mother involved, 0-otherwise | 1/21 |
|  |  |  |  |
| **Freedom of Movement** | Within last 6 months have you gone to the market? | 2-alone, 1-at all, 0-no | 1/18 |
|  | Within last 6 months have you gone outside the village? | 2-alone, 1-at all, 0-no | 1/18 |
|  | Within last 6 months have you gone to a paternal home/friends/relatives? | 2-alone, 1-at all, 0-no | 1/18 |
|  |  |  |  |
| **Control Over Assets** | When you want or need to buy things like food or clothing for yourself or your family, which of the following answers best describes your situation over the last 6 months? | 1 - mother has own money and can usually buy what she needs OR she occasionally has to get the money from her husband or someone else in the household, 0-otherwise | 1/15 |
|  | If you earn, who keeps your earning? | 1-mother, 0-other | 1/15 |
|  | Do you get to spend your own money independently? | 1-yes, 0-no | 1/15 |
|  | Do you have any inherited property in your possession? | 1-yes, 0-no | 1/15 |
|  | In the last month, did you yourself buy any goods in the market? | 1-yes, 0-no | 1/15 |

Table S2. Components of the Water/sanitation, Assets, Maternal education, and Income (WAMI) index

|  | **Description** | **Range** | |  |
| --- | --- | --- | --- | --- |
| ***Water/sanitation*** | Using World Health Organization definitions* of access to improved water and improved sanitation, households with access to improved water or improved sanitation are assigned a score of 4 for each. Households without access to improved water or improved sanitation are assigned a score of 0 for each. These scores were summed. | 0-8 | |  |
|  |  |  |  |  |
|  |  |  |  |  |
| ***Assets*** | Eight priority assets were selected using random forests with ≥1 antenatal care visit as the outcome. For each asset, households were assigned a 1 if they have the asset and 0 if they do not have the asset. These scores were summed. | 0-8 | |  |
|  |  |  |  |  |
|  |  |  |  |  |
| ***Maternal education*** | Each child’s mother provided the number of years of schooling she had completed, ranging from 0 to 16 years. This number was divided by 2. | 0-8 | |  |
|  |  |  |  |  |
| ***Income*** | Monthly household income was converted to US dollars using the exchange rate from January 1, 2020. Income was divided into octiles using the following scores and cutoffs: 1 (0–26), 2 (26.01-47), 3 (47.01-72), 4 (72.01-106), 5 (106.01-135), 6 (135.01-200), 7 (200.01-293), 8 (293+). | 0-8 | |  |
|  |  |  |  |  |
|  |  |  |  |  |
| ***TOTAL*** | Scores in water and sanitation, assets, mother’s education, and income were summed then divided by 32. | 0-1 |  |  |
| *(WHO/UNICEF Joint Monitoring Program for Water Supply, Sanitation and Hygiene (JMP), 2021a, 2021b) | | | |  |

Table S3. Population average treatment effects of overall empowerment for all pairwise comparisons of the categorical antenatal care (ANC) measure, full sample

| ANC outcome | Empowerment group | ATE | 95% CI |
| --- | --- | --- | --- |
| ≥4 ANC vs. 1-3 ANC (n=1391) | High vs. low | **0.15** | 0.06, 0.24 |
|  | High vs. medium | **0.09** | 0.03, 0.16 |
|  | Medium vs. low | 0.06 | -0.01, 0.13 |
| no ANC vs. 1-3 ANC (n=1059) | High vs. low | -0.07 | -0.14, 0.01 |
|  | High vs. medium | -0.03 | -0.09, 0.03 |
|  | Medium vs. low | -0.04 | -0.10, 0.03 |
| ≥4 ANC vs. no ANC (n=768) | High vs. low | **0.15** | 0.05, 0.25 |
|  | High vs. medium | **0.08** | 0.01, 0.15 |
|  | Medium vs. low | 0.07 | -0.01, 0.16 |
| Models control for women’s age (years), women’s depressive symptoms (CESD), women’s education (years), education differential between the woman and her partner (years), child’s age (months), number of members, number of children under 15, and household wealth. Standard errors clustered at the village level. | | | |

Table S4. Population average treatment effects of overall empowerment on number of antenatal care (ANC) visits, full and stratified samples

| ANC outcome | Empowerment group | WAMI tercile | ATE | 95% CI |
| --- | --- | --- | --- | --- |
| Number of ANC (n=1609) | High vs. low |  | **1.07** | 0.67, 1.47 |
|  | High vs. medium |  | **0.53** | 0.19, 0.87 |
|  | Medium vs. low |  | **0.54** | 0.25, 0.834 |
|  |  |  |  |  |
| Number of ANC (n=1609) | High vs. low | Bottom third | **1.04** | 0.43, 1.66 |
|  |  | Middle third | **1.06** | 0.45, 1.67 |
|  |  | Upper third | **1.11** | 0.45, 1.77 |
|  | High vs. medium | Bottom third | **0.53** | 0.05, 1.00 |
|  |  | Middle third | 0.53 | -0.04, 1.10 |
|  |  | Upper third | 0.53 | -0.14, 1.19 |
|  | Medium vs. low | Bottom third | **0.52** | 0.04, 1.00 |
|  |  | Middle third | **0.54** | 0.03, 1.04 |
|  |  | Upper third | **0.58** | 0.11, 1.06 |
| Models control for women’s age (years), women’s depressive symptoms (CESD), women’s education (years), education differential between the woman and her partner (years), child’s age (months), number of members, number of children under 15, and household wealth. Standard errors clustered at the village level. | | | | |

Table S5. Population average treatment effects of overall empowerment for all pairwise comparisons of the categorical antenatal care (ANC) measure, stratified by WAMI tercile

| ANC outcome | Empowerment group | WAMI tercile | ATE | 95% CI |
| --- | --- | --- | --- | --- |
| ≥4 ANC vs. 1-3 ANC (n=1391) | High vs. low | Bottom third | 0.14 | -0.00, 0.28 |
|  |  | Middle third | **0.15** | 0.00, 0.30 |
|  |  | Upper third | **0.17** | 0.03, 0.30 |
|  | High vs. medium | Bottom third | 0.09 | -0.01, 0.18 |
|  |  | Middle third | 0.09 | -0.02, 0.20 |
|  |  | Upper third | 0.10 | -0.02, 0.21 |
|  | Medium vs. low | Bottom third | 0.06 | -0.07, 0.18 |
|  |  | Middle third | 0.06 | -0.06, 0.18 |
|  |  | Upper third | 0.07 | -0.03, 0.17 |
|  |  |  |  |  |
| no ANC vs. 1-3 ANC (n=1059) | High vs. low | Bottom third | -0.07 | -0.21, 0.06 |
|  |  | Middle third | -0.07 | -0.18, 0.05 |
|  |  | Upper third | -0.05 | -0.14, 0.04 |
|  | High vs. medium | Bottom third | -0.03 | -0.15, 0.08 |
|  |  | Middle third | -0.03 | -0.13, 0.07 |
|  |  | Upper third | -0.02 | -0.11, 0.06 |
|  | Medium vs. low | Bottom third | -0.04 | -0.16, 0.08 |
|  |  | Middle third | -0.03 | -0.13, 0.06 |
|  |  | Upper third | -0.03 | -0.12, 0.06 |
|  |  |  |  |  |
| ≥4 ANC vs. no ANC (n=768) | High vs. low | Bottom third | 0.18 | -0.04, 0.41 |
|  |  | Middle third | 0.17 | -0.03, 0.36 |
|  |  | Upper third | **0.10** | 0.02, 0.18 |
|  | High vs. medium | Bottom third | 0.10 | -0.06, 0.26 |
|  |  | Middle third | 0.09 | -0.05, 0.22 |
|  |  | Upper third | 0.05 | -0.02, 0.11 |
|  | Medium vs. low | Bottom third | 0.09 | -0.12, 0.29 |
|  |  | Middle third | 0.08 | -0.08, 0.24 |
|  |  | Upper third | 0.05 | -0.03, 0.14 |
| Models control for women’s age (years), women’s depressive symptoms (CESD), women’s education (years), education differential between the woman and her partner (years), child’s age (months), number of members, number of children under 15, and household wealth. Standard errors clustered at the village level. | | | | |

Table S6. Population average treatment effects of decision-making power for all pairwise comparisons of the categorical antenatal care (ANC) measure, full sample

| ANC outcome | Empowerment group | ATE | 95% CI |
| --- | --- | --- | --- |
| ≥4 ANC vs. 1-3 ANC (n=1391) | High vs. low | **0.15** | 0.06, 0.24 |
|  | High vs. medium | 0.06 | -0.01, 0.13 |
|  | Medium vs. low | **0.09** | 0.01, 0.17 |
| no ANC vs. 1-3 ANC (n=1059) | High vs. low | -0.07 | -0.15, 0.01 |
|  | High vs. medium | **-0.08** | -0.13, -0.03 |
|  | Medium vs. low | 0.01 | -0.06, 0.09 |
| ≥4 ANC vs. no ANC (n=768) | High vs. low | **0.14** | 0.02, 0.25 |
|  | High vs. medium | **0.09** | 0.02, 0.16 |
|  | Medium vs. low | 0.05 | -0.06, 0.16 |
| Models control for women’s age (years), women’s depressive symptoms (CESD), women’s education (years), education differential between the woman and her partner (years), child’s age (months), number of members, number of children under 15, and household wealth. Standard errors clustered at the village level. | | | |

Table S7. Population average treatment effects of freedom of movement for all pairwise comparisons of the categorical antenatal care (ANC) measure, full sample

| ANC outcome | Empowerment group | ATE | 95% CI |
| --- | --- | --- | --- |
| ≥4 ANC vs. 1-3 ANC (n=1391) | High vs. low | 0.04 | -0.05, 0.12 |
|  | High vs. medium | 0.04 | -0.04, 0.11 |
|  | Medium vs. low | -0.00 | -0.07, 0.06 |
| no ANC vs. 1-3 ANC (n=1059) | High vs. low | 0.01 | -0.07, 0.09 |
|  | High vs. medium | 0.05 | -0.02, 0.11 |
|  | Medium vs. low | -0.04 | -0.11, 0.04 |
| ≥4 ANC vs. no ANC (n=768) | High vs. low | 0.02 | -0.11, 0.15 |
|  | High vs. medium | -0.02 | -0.10, 0.06 |
|  | Medium vs. low | 0.04 | -0.06, 0.14 |
| Models control for women’s age (years), women’s depressive symptoms (CESD), women’s education (years), education differential between the woman and her partner (years), child’s age (months), number of members, number of children under 15, and household wealth. Standard errors clustered at the village level. | | | |

Table S8. Population average treatment effects of control over assets for all pairwise comparisons of the categorical antenatal care (ANC) measure, full sample

| ANC outcome | Empowerment group | ATE | 95% CI |
| --- | --- | --- | --- |
| ≥4 ANC vs. 1-3 ANC (n=1391) | High vs. low | **0.15** | 0.07, 0.23 |
|  | High vs. medium | **0.11** | 0.05, 0.18 |
|  | Medium vs. low | 0.04 | -0.03, 0.11 |
| no ANC vs. 1-3 ANC (n=1059) | High vs. low | -0.05 | -0.13, 0.03 |
|  | High vs. medium | -0.02 | -0.09, 0.06 |
|  | Medium vs. low | -0.03 | -0.09, 0.03 |
| ≥4 ANC vs. no ANC (n=768) | High vs. low | **0.13** | 0.03, 0.24 |
|  | High vs. medium | 0.10 | -0.00, 0.21 |
|  | Medium vs. low | 0.03 | -0.05, 0.11 |
| Models control for women’s age (years), women’s depressive symptoms (CESD), women’s education (years), education differential between the woman and her partner (years), child’s age (months), number of members, number of children under 15, and household wealth. Standard errors clustered at the village level. | | | |

Figure S1. Population average treatment effects of decision-making power for all pairwise comparisons of the categorical antenatal care (ANC) measure, stratified by WAMI tercile


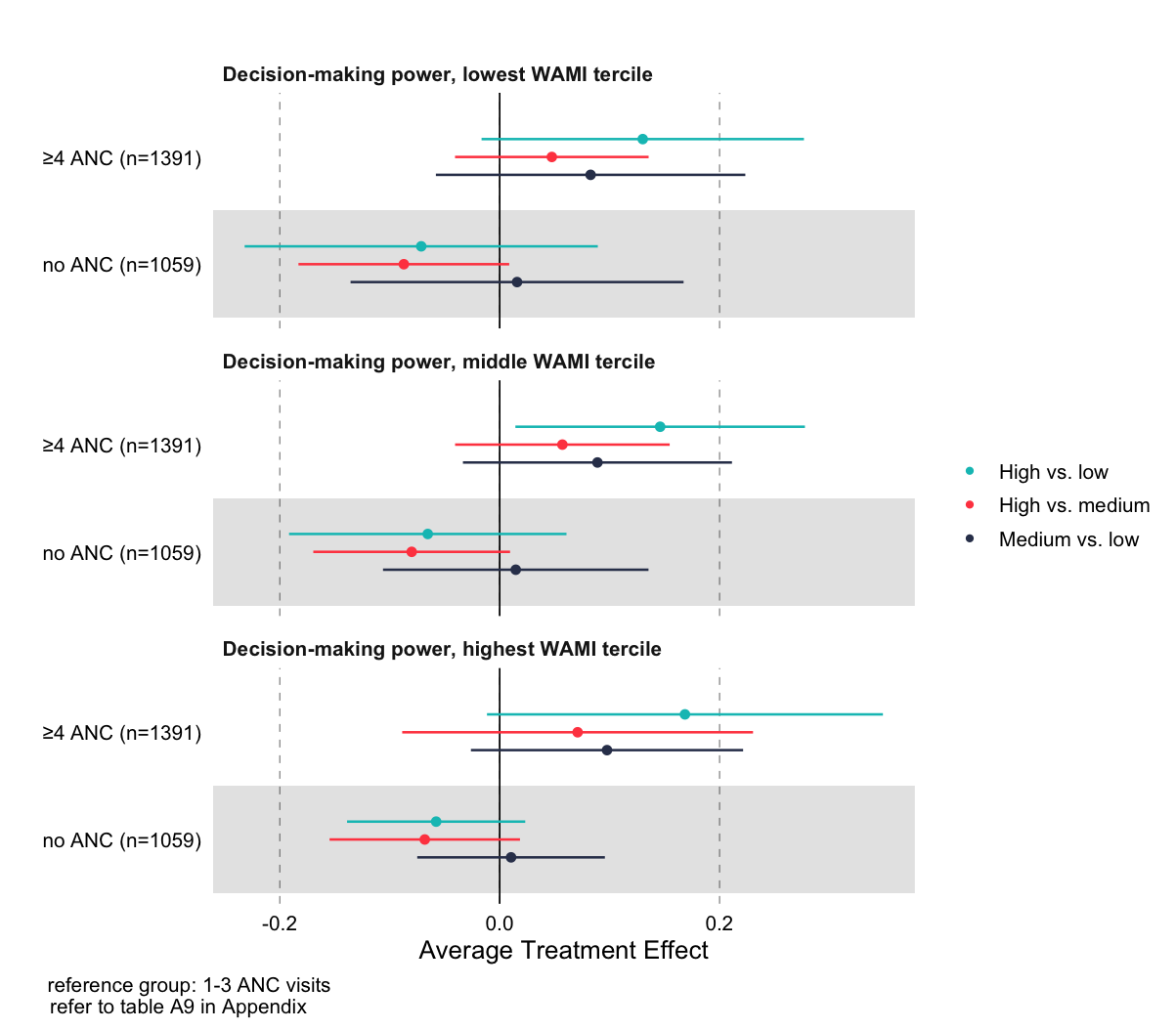


Table S9. Population average treatment effects of decision-making power for all pairwise comparisons of the categorical antenatal care (ANC) measure, stratified by WAMI tercile

| ANC outcome | Empowerment group | WAMI tercile | ATE | 95% CI |
| --- | --- | --- | --- | --- |
| ≥4 ANC vs. 1-3 ANC (n=1391) | High vs. low | Bottom third | 0.13 | -0.02, 0.28 |
|  |  | Middle third | **0.15** | 0.01, 0.28 |
|  |  | Upper third | 0.17 | -0.01, 0.35 |
|  | High vs. medium | Bottom third | 0.05 | -0.04, 0.14 |
|  |  | Middle third | 0.06 | -0.04, 0.16 |
|  |  | Upper third | 0.07 | -0.09, 0.23 |
|  | Medium vs. low | Bottom third | 0.08 | -0.06, 0.22 |
|  |  | Middle third | 0.09 | -0.03, 0.21 |
|  |  | Upper third | 0.10 | -0.03, 0.22 |
|  |  |  |  |  |
| no ANC vs. 1-3 ANC (n=1059) | High vs. low | Bottom third | -0.07 | -0.23, 0.09 |
|  |  | Middle third | -0.07 | -0.19, 0.06 |
|  |  | Upper third | -0.06 | -0.14, 0.02 |
|  | High vs. medium | Bottom third | -0.09 | -0.18, 0.01 |
|  |  | Middle third | -0.08 | -0.17, 0.01 |
|  |  | Upper third | -0.07 | -0.16, 0.02 |
|  | Medium vs. low | Bottom third | 0.02 | -0.14, 0.17 |
|  |  | Middle third | 0.02 | -0.11, 0.14 |
|  |  | Upper third | 0.01 | -0.08, 0.10 |
|  |  |  |  |  |
| ≥4 ANC vs. no ANC (n=768) | High vs. low | Bottom third | 0.17 | -0.09, 0.43 |
|  |  | Middle third | 0.15 | -0.08, 0.39 |
|  |  | Upper third | 0.09 | -0.04, 0.22 |
|  | High vs. medium | Bottom third | 0.11 | -0.04, 0.27 |
|  |  | Middle third | 0.10 | -0.04, 0.24 |
|  |  | Upper third | 0.06 | -0.01, 0.12 |
|  | Medium vs. low | Bottom third | 0.06 | -0.20, 0.32 |
|  |  | Middle third | 0.06 | -0.15, 0.26 |
|  |  | Upper third | 0.04 | -0.09, 0.16 |
| Models control for women’s age (years), women’s depressive symptoms (CESD), women’s education (years), education differential between the woman and her partner (years), child’s age (months), number of members, number of children under 15, and household wealth. Standard errors clustered at the village level. | | | | |

Figure S2. Population average treatment effects of freedom of movement for all pairwise comparisons of the categorical antenatal care (ANC) measure, stratified by WAMI tercile


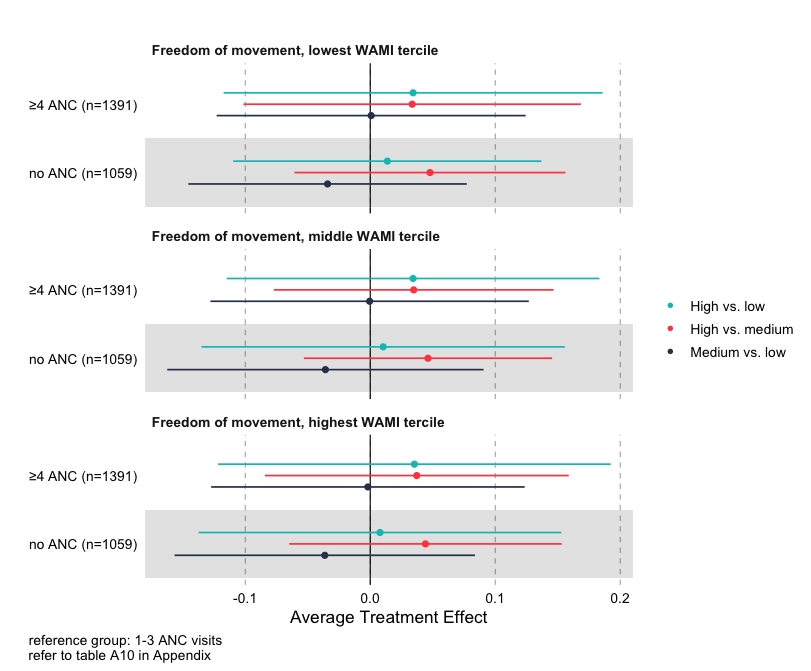


Table S10. Population average treatment effects of freedom of movement for all pairwise comparisons of the categorical antenatal care (ANC) measure, stratified by WAMI tercile

| ANC outcome | Empowerment group | WAMI tercile | ATE | 95% CI |  |
| --- | --- | --- | --- | --- | --- |
| ≥4 ANC vs. 1-3 ANC (n=1391) | High vs. low | Bottom third | 0.03 | -0.12, 0.19 |  |
|  |  | Middle third | 0.03 | -0.12, 0.18 |  |
|  |  | Upper third | 0.04 | -0.12, 0.19 |  |
|  | High vs. medium | Bottom third | 0.03 | -0.10, 0.17 |  |
|  |  | Middle third | 0.04 | -0.08, 0.15 |  |
|  |  | Upper third | 0.04 | -0.08, 0.16 |  |
|  | Medium vs. low | Bottom third | 0.00 | -0.12, 0.12 |  |
|  |  | Middle third | 0.00 | -0.13, 0.13 |  |
|  |  | Upper third | -0.00 | -0.13, 0.12 |  |
|  |  |  |  |  |  |
| no ANC vs. 1-3 ANC (n=1059) | High vs. low | Bottom third | 0.01 | -0.11, 0.14 | |
|  |  | Middle third | 0.01 | -0.14, 0.16 | |
|  |  | Upper third | 0.01 | -0.14, 0.15 | |
|  | High vs. medium | Bottom third | 0.05 | -0.06, 0.16 | |
|  |  | Middle third | 0.05 | -0.05, 0.15 | |
|  |  | Upper third | 0.04 | -0.07, 0.15 | |
|  | Medium vs. low | Bottom third | -0.03 | -0.15, 0.08 | |
|  |  | Middle third | -0.04 | -0.16, 0.09 | |
|  |  | Upper third | -0.04 | -0.16, 0.08 | |
|  |  |  |  |  |  |
| ≥4 ANC vs. no ANC (n=768) | High vs. low | Bottom third | 0.02 | -0.23, 0.27 |  |
|  |  | Middle third | 0.02 | -0.21, 0.26 |  |
|  |  | Upper third | 0.01 | -0.12, 0.15 |  |
|  | High vs. medium | Bottom third | -0.03 | -0.21, 0.16 |  |
|  |  | Middle third | -0.02 | -0.17, 0.13 |  |
|  |  | Upper third | -0.01 | -0.10, 0.07 |  |
|  | Medium vs. low | Bottom third | 0.05 | -0.16, 0.26 |  |
|  |  | Middle third | 0.05 | -0.15, 0.24 |  |
|  |  | Upper third | 0.03 | -0.10, 0.15 |  |
| Models control for women’s age (years), women’s depressive symptoms (CESD), women’s education (years), education differential between the woman and her partner (years), child’s age (months), number of members, number of children under 15, and household wealth. Standard errors clustered at the village level. | | | | |  |

Figure S3. Population average treatment effects of control over assets for all pairwise comparisons of the categorical antenatal care (ANC) measure, stratified by WAMI tercile


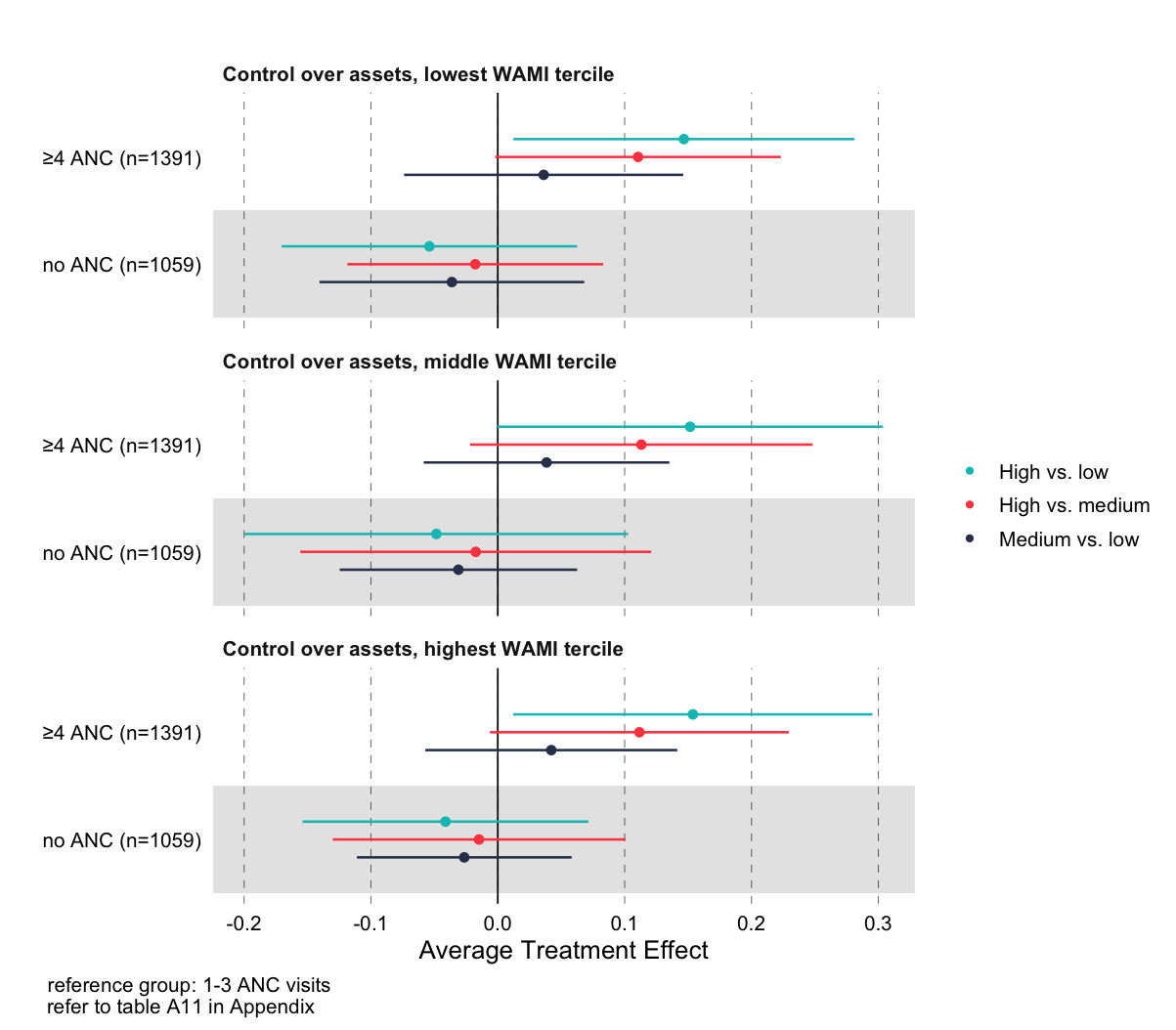


Table S11. Population average treatment effects of control over assets for all pairwise comparisons of the categorical antenatal care (ANC) measure, stratified by WAMI tercile

| ANC outcome | Empowerment group | WAMI tercile | ATE | 95% CI |
| --- | --- | --- | --- | --- |
| ≥4 ANC vs. 1-3 ANC (n=1391) | High vs. low | Bottom third | **0.15** | 0.01, 0.28 |
|  |  | Middle third | 0.15 | -0.00, 0.30 |
|  |  | Upper third | **0.15** | 0.01, 0.30 |
|  | High vs. medium | Bottom third | 0.11 | -0.00, 0.22 |
|  |  | Middle third | 0.11 | -0.02, 0.25 |
|  |  | Upper third | 0.11 | -0.01, 0.23 |
|  | Medium vs. low | Bottom third | 0.04 | -0.07, 0.15 |
|  |  | Middle third | 0.04 | -0.06, 0.14 |
|  |  | Upper third | 0.04 | -0.06, 0.14 |
|  |  |  |  |  |
| no ANC vs. 1-3 ANC (n=1059) | High vs. low | Bottom third | -0.05 | -0.17, 0.06 |
|  |  | Middle third | -0.03 | -0.20, 0.10 |
|  |  | Upper third | -0.04 | -0.15, 0.07 |
|  | High vs. medium | Bottom third | -0.02 | -0.12, 0.08 |
|  |  | Middle third | -0.02 | -0.16, 0.12 |
|  |  | Upper third | -0.02 | -0.13, 0.10 |
|  | Medium vs. low | Bottom third | -0.04 | -0.14, 0.07 |
|  |  | Middle third | -0.03 | -0.13, 0.06 |
|  |  | Upper third | -0.03 | -0.11, 0.06 |
|  |  |  |  |  |
| ≥4 ANC vs. no ANC (n=768) | High vs. low | Bottom third | 0.18 | -0.01, 0.36 |
|  |  | Middle third | 0.15 | -0.06, 0.36 |
|  |  | Upper third | 0.08 | -0.02, 0.18 |
|  | High vs. medium | Bottom third | 0.13 | -0.03, 0.30 |
|  |  | Middle third | 0.11 | -0.09, 0.32 |
|  |  | Upper third | 0.06 | -0.02, 0.14 |
|  | Medium vs. low | Bottom third | 0.04 | -0.15, 0.24 |
|  |  | Middle third | 0.04 | -0.10, 0.18 |
|  |  | Upper third | 0.02 | -0.07, 0.11 |
| Models control for women’s age (years), women’s depressive symptoms (CESD), women’s education (years), education differential between the woman and her partner (years), child’s age (months), number of members, number of children under 15, and household wealth. Standard errors clustered at the village level. | | | | |

Table S12. Population average treatment effects of decision-making power on number of antenatal care (ANC) visits, full and stratified samples

| ANC outcome | Empowerment group | WAMI tercile | ATE | 95% CI |
| --- | --- | --- | --- | --- |
| Number of ANC (n=1609) | High vs. low |  | **0.76** | 0.35, 1.16 |
|  | High vs. medium |  | **0.36** | 0.07, 0.65 |
|  | Medium vs. low |  | **0.40** | 0.02, 0.77 |
|  |  |  |  |  |
| Number of ANC (n=1609) | High vs. low | Bottom third | **0.71** | 0.05, 1.38 |
|  |  | Middle third | **0.74** | 0.16, 1.33 |
|  |  | Upper third | **0.82** | 0.02, 1.62 |
|  | High vs. medium | Bottom third | 0.33 | -0.07, 0.74 |
|  |  | Middle third | 0.35 | -0.10, 0.81 |
|  |  | Upper third | 0.40 | -0.26, 1.07 |
|  | Medium vs. low | Bottom third | 0.38 | -0.24, 1.00 |
|  |  | Middle third | 0.39 | -0.14, 0.92 |
|  |  | Upper third | 0.42 | -0.30, 1.13 |
| Models control for women’s age (years), women’s depressive symptoms (CESD), women’s education (years), education differential between the woman and her partner (years), child’s age (months), number of members, number of children under 15, and household wealth. Standard errors clustered at the village level. | | | | |

Table S13. Population average treatment effects of freedom of movement on number of antenatal care (ANC) visits, full and stratified samples

| ANC outcome | Empowerment group | WAMI tercile | ATE | 95% CI |
| --- | --- | --- | --- | --- |
| Number of ANC (n=1609) | High vs. low |  | 0.30 | -0.16, 0.77 |
|  | High vs. medium |  | 0.04 | -0.32, 0.40 |
|  | Medium vs. low |  | 0.26 | -0.06, 0.59 |
|  |  |  |  |  |
| Number of ANC (n=1609) | High vs. low | Bottom third | 0.29 | -0.36, 0.93 |
|  |  | Middle third | 0.20 | -0.39, 0.98 |
|  |  | Upper third | 0.33 | -0.48, 1.13 |
|  | High vs. medium | Bottom third | 0.03 | -0.51, 0.58 |
|  |  | Middle third | 0.04 | -0.51, 0.59 |
|  |  | Upper third | 0.05 | -0.57, 0.67 |
|  | Medium vs. low | Bottom third | 0.25 | -0.28, 0.78 |
|  |  | Middle third | 0.26 | -0.31, 0.82 |
|  |  | Upper third | 0.28 | -0.33, 0.89 |
| Models control for women’s age (years), women’s depressive symptoms (CESD), women’s education (years), education differential between the woman and her partner (years), child’s age (months), number of members, number of children under 15, and household wealth. Standard errors clustered at the village level. | | | | |

Table S14. Population average treatment effects of control over assets on number of antenatal care (ANC) visits, full and stratified samples

| ANC outcome | Empowerment group | WAMI tercile | ATE | 95% CI |
| --- | --- | --- | --- | --- |
| Number of ANC (n=1609) | High vs. low |  | **0.88** | 0.43, 1.34 |
|  | High vs. medium |  | **0.59** | 0.19, 1.00 |
|  | Medium vs. low |  | 0.29 | -0.03, 0.62 |
|  |  |  |  |  |
| Number of ANC (n=1609) | High vs. low | Bottom third | **0.89** | **0.28, 1.49** |
|  |  | Middle third | **0.88** | **0.11, 1.66** |
|  |  | Upper third | 0.88 | -0.06, 1.82 |
|  | High vs. medium | Bottom third | 0.59 | 0.03, 1.15 |
|  |  | Middle third | 0.59 | -0.13, 1.30 |
|  |  | Upper third | 0.60 | -0.20, 1.39 |
|  | Medium vs. low | Bottom third | 0.30 | -0.15, 0.74 |
|  |  | Middle third | 0.29 | -0.17, 0.76 |
|  |  | Upper third | 0.29 | -0.32, 0.89 |
| Models control for women’s age (years), women’s depressive symptoms (CESD), women’s education (years), education differential between the woman and her partner (years), child’s age (months), number of members, number of children under 15, and household wealth. Standard errors clustered at the village level. | | | | |

Figure S4. Population average treatment effects of overall empowerment for all pairwise comparisons of the categorical antenatal care (ANC) measure, stratified by age of mother


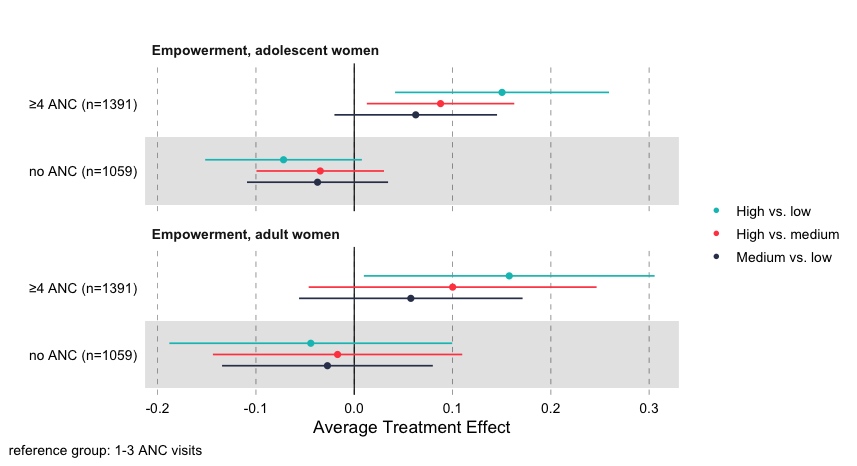


Figure S5. Population average treatment effects of overall empowerment for all pairwise comparisons of the categorical antenatal care (ANC) measure, stratified by presence of other children under 15


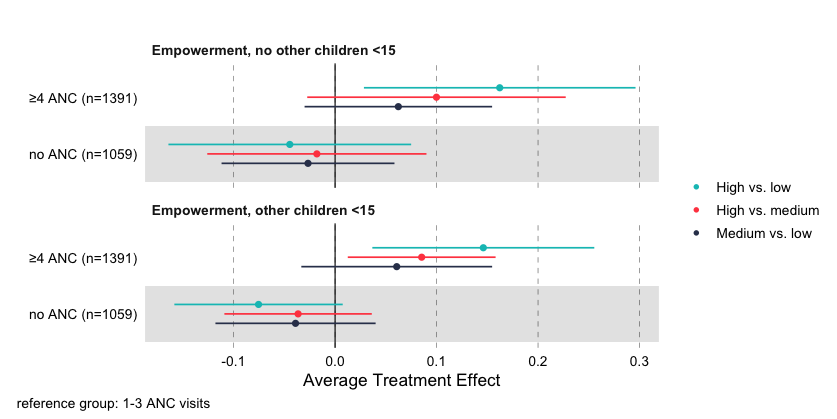


Figure S6. Population average treatment effects of overall empowerment for all pairwise comparisons of the categorical antenatal care (ANC) measure, stratified by presence of in-laws


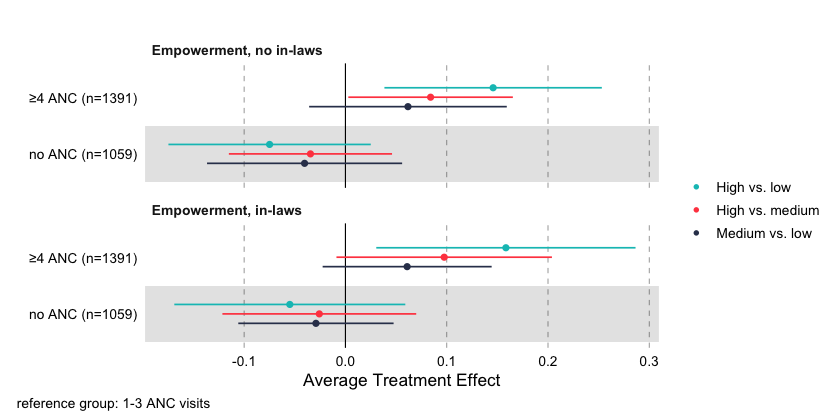


Figure S7. Population average treatment effects of overall empowerment for all pairwise comparisons of the categorical antenatal care (ANC) measure, stratified by mother’s depressive symptoms


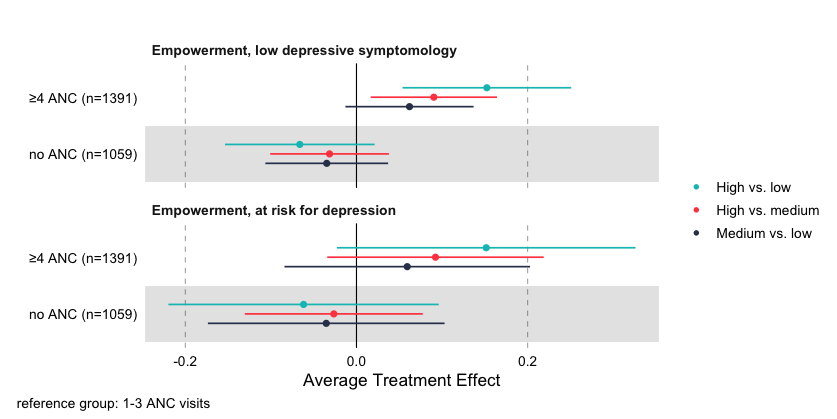


Table S15. Population average treatment effects of overall empowerment (estimated with parametric G-computation) for all pairwise comparisons of the categorical antenatal care (ANC) measure, full sample

| ANC outcome | Empowerment group | ATE | 95% CI |
| --- | --- | --- | --- |
| ≥4 ANC vs. 1-3 ANC (n=1391) | High vs. low | **0.15** | 0.07, 0.27 |
|  | High vs. medium | **0.09** | 0.04, 0.15 |
|  | Medium vs. low | **0.06** | 0.03, 0.12 |
| no ANC vs. 1-3 ANC (n=1059) | High vs. low | -0.08 | -0.13, 0.02 |
|  | High vs. medium | -0.05 | -0.07, 0.02 |
|  | Medium vs. low | -0.03 | -0.07, 0.01 |
| ≥4 ANC vs. no ANC (n=768) | High vs. low | **0.15** | 0.13, 0.21 |
|  | High vs. medium | **0.09** | 0.00, 0.16 |
|  | Medium vs. low | **0.06** | 0.03, 0.21 |
| Models control for women’s age (years), women’s depressive symptoms (CESD), women’s education (years), education differential between the woman and her partner (years), child’s age (months), number of members, number of children under 15, and household wealth. Standard errors clustered at the village level. | | | |

Table S16. Components and loadings for Survey-based Women’s emPowERment (SWPER) Index

| **Variable** | **PC1: Decision making power** | **PC2: Freedom of movement** | **PC3: Control over assets** |
| --- | --- | --- | --- |
| Went to market in last 6 months | 0.02 | **0.43** | 0.18 |
| Went outside village in last 6 months | -0.01 | **0.59** | -0.06 |
| Went to friend’s/relative’s home in last 6 months | -0.02 | **0.56** | -0.10 |
| Has her own money and can usually buy what she needs | 0.03 | 0.03 | **0.47** |
| Keeps her own earnings | 0.01 | -0.02 | **0.59** |
| Can spend her own money independently | 0.00 | -0.06 | **0.59** |
| Possesses inherited property | 0.00 | -0.06 | **0.05** |
| Bought herself goods in the last month | 0.05 | 0.37 | **0.14** |
| Involved in decisions about food purchases | **0.38** | -0.00 | 0.05 |
| Involved in decisions about child education | **0.33** | 0.01 | -0.05 |
| Involved in decisions about child health expenses | **0.41** | -0.00 | -0.03 |
| Involved in decisions about mother's health | **0.35** | 0.02 | -0.05 |
| Involved in decisions about household improvements | **0.39** | -0.04 | 0.04 |
| Involved in decisions about household goods | **0.41** | -0.02 | -0.01 |
| Involved in decisions about child's toys, books | **0.37** | 0.02 | 0.02 |

Figure S8. Scree Plot for Survey-based Women’s emPowERment (SWPER) Index


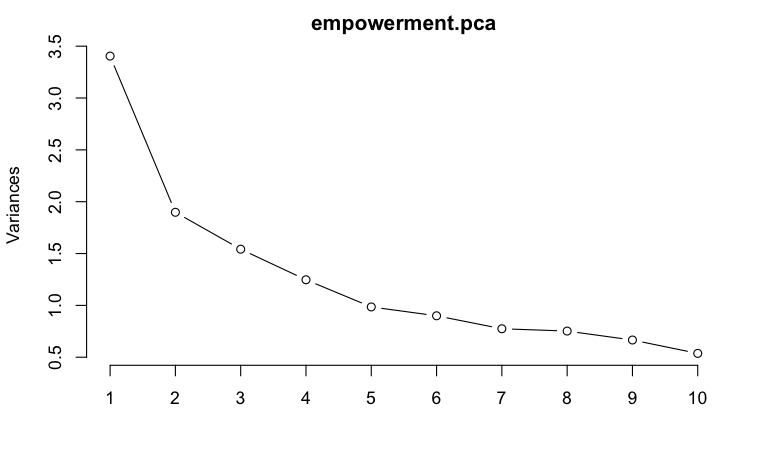


Table S17. Population average treatment effects of decision-making power (SPWER, PC1) for all pairwise comparisons of the categorical antenatal care (ANC) measure, full sample

| ANC outcome | Empowerment group | ATE* | 95% CI^†^ |
| --- | --- | --- | --- |
| ≥4 ANC vs. 1-3 ANC (n=1391) | High vs. low | **0.11** | 0.02, 0.19 |
|  | High vs. medium | **0.08** | 0.01, 0.15 |
|  | Medium vs. low | 0.03 | -0.04, 0.10 |
| no ANC vs. 1-3 ANC (n=1059) | High vs. low | **-0.10** | -0.18, -0.03 |
|  | High vs. medium | **-0.06** | -0.12, -0.01 |
|  | Medium vs. low | -0.04 | -0.10, 0.03 |
| ≥4 ANC vs. no ANC (n=768) | High vs. low | **0.15** | 0.05, 0.24 |
|  | High vs. medium | **0.09** | 0.02, 0.16 |
|  | Medium vs. low | 0.05 | -0.03, 0.13 |
| Models control for women’s age (years), women’s depressive symptoms (CESD), women’s education (years), education differential between the woman and her partner (years), child’s age (months), number of members, number of children under 15, and household wealth. Standard errors clustered at the village level. | | | |

Table S18. Population average treatment effects of freedom of movement (SWPER, PC2) for all pairwise comparisons of the categorical antenatal care (ANC) measure, full sample

| ANC outcome | Empowerment group | ATE | 95% CI |
| --- | --- | --- | --- |
| ≥4 ANC vs. 1-3 ANC (n=1391) | High vs. low | 0.07 | -0.01, 0.14 |
|  | High vs. medium | 0.02 | -0.05, 0.09 |
|  | Medium vs. low | 0.04 | -0.01, 0.10 |
| no ANC vs. 1-3 ANC (n=1059) | High vs. low | 0.02 | -0.06, 0.09 |
|  | High vs. medium | 0.04 | -0.03, 0.10 |
|  | Medium vs. low | -0.02 | -0.07, 0.04 |
| ≥4 ANC vs. no ANC (n=768) | High vs. low | 0.02 | -0.08, 0.13 |
|  | High vs. medium | -0.03 | -0.11, 0.06 |
|  | Medium vs. low | 0.05 | -0.02, 0.12 |
| Models control for women’s age (years), women’s depressive symptoms (CESD), women’s education (years), education differential between the woman and her partner (years), child’s age (months), number of members, number of children under 15, and household wealth. Standard errors clustered at the village level. | | | |

Table S19. Population average treatment effects of control over assets (SWPER, PC3) for all pairwise comparisons of the categorical antenatal care (ANC) measure, full sample

| ANC outcome | Empowerment group | ATE | 95% CI |
| --- | --- | --- | --- |
| ≥4 ANC vs. 1-3 ANC (n=1391) | High vs. low | **0.10** | 0.03, 0.18 |
|  | High vs. medium | **0.11** | 0.05, 0.17 |
|  | Medium vs. low | -0.01 | -0.08, 0.06 |
| no ANC vs. 1-3 ANC (n=1059) | High vs. low | -0.04 | -0.12, 0.03 |
|  | High vs. medium | 0.00 | -0.07, 0.07 |
|  | Medium vs. low | -0.05 | -0.10, 0.01 |
| ≥4 ANC vs. no ANC (n=768) | High vs. low | **0.09** | 0.01, 0.18 |
|  | High vs. medium | 0.08 | -0.00, 0.16 |
|  | Medium vs. low | 0.01 | -0.08, 0.10 |
| Models control for women’s age (years), women’s depressive symptoms (CESD), women’s education (years), education differential between the woman and her partner (years), child’s age (months), number of members, number of children under 15, and household wealth. Standard errors clustered at the village level. | | | |
